# Real-life use of onabotulinumtoxinA reduces healthcare resource utilization in individuals with chronic migraine: the REPOSE study

**DOI:** 10.1186/s10194-021-01260-4

**Published:** 2021-06-02

**Authors:** Katja Kollewe, Charly Gaul, Astrid Gendolla, Katherine Sommer

**Affiliations:** 1 Medical School Hannover, Carl-Neuberg-Straße 1, 30625 Hannover, Germany; 2 Migraine and Headache Clinic, Königstein, Germany; 3 Praxis fur Neurologie, Essen, Germany; 4 Allergan, An AbbVie Company, Marlow, Buckinghamshire UK

**Keywords:** OnabotulinumtoxinA, Chronic migraine, Headache, Economic, Healthcare, Burden, Healthcare resource utilization

## Abstract

**Background:**

Chronic migraine (CM) is associated with substantial economic burden. Real-world data suggests that onabotulinumtoxinA treatment for CM reduces healthcare resource utilisation (HRU) and related costs.

**Methods:**

REPOSE was a 2-year prospective, multicentre, non-interventional, observational study to describe the real-world use of onabotulinumtoxinA in adult patients with CM. This analysis examined the impact of onabotulinumtoxinA on HRU. Patients received onabotulinumtoxinA treatment approximately every 12 weeks according to their physicians’ discretion, guided by the summary of product characteristics (SPC) and PREEMPT injection paradigm. HRU outcome measures were collected at baseline and all administration visits and included headache-related hospitalizations and healthcare professional (HCP) visits. Health economic data, including family doctor and specialist visits, inpatient treatment for headache, acupuncture, technical diagnostics, use of nonpharmacologic remedies, and work productivity were also collected for patients enrolled at German study centres.

**Results:**

Overall, 641 patients were enrolled at 78 study centres across 7 countries (Germany, UK, Italy, Spain, Norway, Sweden, and Russia), 633 received ≥1 onabotulinumtoxinA dose, and 128 completed the 2-year study. Patients were, on average, aged 45 years, 85% were female, and 60% (*n* = 377) were from Germany. At the end of the 2-year observation period, significantly fewer patients reported headache-related hospitalizations (*p* < 0.02) and HCP visits (*p* < 0.001) within the past 3 months than in the 3 months before baseline. In the German population, reductions were observed across all health services at all follow-up visits compared with baseline. The percentage of patients who saw a family doctor decreased from 41.7% at baseline to 13.5% at administration visit 8 and visits to a medical specialist decreased from 61.7% to 5.2% of patients. Inpatient acute treatment and technical diagnostics declined from 6.4% and 19.7% of patients at baseline to 0.0% and 1.0% at administration 8, respectively. The use of nonpharmacologic remedies and medication for the acute treatment of migraine also decreased with continued onabotulinumtoxinA treatment. Work incapacity, disability, absenteeism, and impaired performance at school/work improved with onabotulinumtoxinA treatment for CM over the 2-year observation period.

**Conclusions:**

Real-world evidence from REPOSE demonstrates that onabotulinumtoxinA treatment is associated with decreased HRU and supports the long-term benefits associated with the use of onabotulinumtoxinA for CM in clinical practice.

**Trial registration:**

NCT01686581. Name of registry: ClinicalTrials.gov. URL of registry: Date of retrospective registration: September 18, 2012. Date of enrolment of first patient: July 23, 2012.

**Supplementary Information:**

The online version contains supplementary material available at 10.1186/s10194-021-01260-4.

## Background

Migraine is one of the most common neurological diseases. Chronic migraine (CM) is defined as ≥15 headache days/month for more than 3 months, from which a minimum of 8 days/month fulfill the criteria for migraine [1]. The global prevalence rates of CM migraine range from 1.4–2.2% [2] and a population-based German Headache Consortium Study estimated the prevalence of CM in Germany at 1.9% [3]. Individuals living with CM often experience a diminished quality of life and societal and familial burden resulting from frequent, incapacitating migraine attacks [4, 5]. Further, CM is associated with substantial disability, healthcare resource utilisation (HRU), and economic burden [4, 6–9].

OnabotulinumtoxinA (BOTOX®; Allergan, an AbbVie Company) is indicated for the preventive therapy of migraine in adult patients with CM and has been shown to reduce HRU in the United States and Europe [10, 11]. The PREEMPT phase 3 clinical trial program provided evidence of the efficacy and safety of onabotulinumtoxinA for headache prevention [12, 13] and these findings are well supported by both clinical and real-world studies [14–19]. Additionally, cost modeling, using patient data and dosing protocols from the PREEMPT clinical trials, EuroQol 5-Dimension Questionnaire (EQ-5D) utility estimates from the REPOSE study, and resource utilization estimates the International Burden of Migraine Study (IBMS) has demonstrated that onabotulinumtoxinA is likely to represent a cost-effective resource in the UK [20]. To establish the complete treatment benefit across different countries and healthcare systems, additional real-world data on the economic impact of onabotulinumtoxinA treatment for CM is needed.

The REal-life use of botulinum toxin for the symptomatic treatment of adults with chronic migraine, measuring healthcare resource utilisation, and Patient-reported OutcomeS observed in practicE (REPOSE) study was a 2-year, multicenter, prospective, observational, open-label study to describe the long term, real-world use of onabotulinumtoxinA for the symptomatic treatment of adults with chronic migraine in Europe. The patient- and physician-reported outcomes from the REPOSE study demonstrated a sustained reduction in headache-day frequency and significant improvement in quality of life measures with onabotulinumtoxinA treatment for CM [17]. This analysis examines HRU utilisation in the REPOSE study with an emphasis on a subset of the German study population that completed an additional health economic questionnaire, which included utilisation of health services and productivity metrics.

## Methods

### Study design

The design of the REPOSE study (NCT01686581) has previously been published in detail [21]. Briefly, REPOSE was a 2-year, prospective, non-interventional, observational, open-label study of patients prescribed onabotulinumtoxinA for the treatment of CM. The REPOSE study was conducted from July 2012 through October 2016 across 78 study centres in 7 countries: Germany, UK, Italy, Spain, Norway/Sweden, and Russia. To collect data on the use of onabotulinumtoxinA in real-life clinical settings, consecutive patients for whom physicians prescribed onabotulinumtoxinA were considered for inclusion in the study. All procedures were performed at the discretion of the physicians according to their clinical judgment and the local standard of medical care.

Eligible patients included adult men and women ≥18 years of age prescribed onabotulinumtoxinA for the treatment of CM. Patients were ineligible if they had received any botulinum toxin serotype within 26 weeks of study enrollment, were concurrently participating in Allergan’s BOTOX® CM Post-Authorisation Safety Study (PASS), or were contraindicated for treatment with onabotulinumtoxinA. Investigators were to refer to the Summary of Product Characteristics (SPC) for information on contraindications, warnings, and pregnancy and lactation. Patients were not excluded for receiving acute or other preventive treatments before study enrollment and were permitted to continue these treatments, as needed, during the study. All patients provided written informed consent before enrollment.

### OnabotulinumtoxinA treatment

OnabotulinumtoxinA was administered according to the physician’s discretion and guided by the SPC and the PREEMPT study protocol [15], which recommends administration of 155 U spread over 31 injection sites at a dosing interval of 12 weeks. In accordance with the SPC, the administration of an additional 40 U over 8 injection sites according to the follow-the-pain strategy to a maximum total dose of 195 U was possible by physician discretion [16]. All procedures were performed according to study physicians’ clinical judgment and the local standard of medical care; physicians were recommended but not required to follow the PREEMPT paradigm. All study investigators obtained ethical approval from their respective ethics committees prior to study initiation. REPOSE was conducted in accordance with the International Conference on Harmonisation Guideline for Good Clinical Practice.

### Outcomes measures

At baseline (administration visit 1), patient demographics, medical history, headache history, and previous/concomitant headache treatment(s) were documented. Treatment effectiveness was measured by the change from baseline in patient estimates of the frequency of headache days, the Migraine Specific Quality of Life Questionnaire (MSQ) v2.1 [22], and EuroQol 5-Dimension Questionnaire (EQ-5D) [23].

Healthcare resource utilisation was assessed in the overall population at baseline and the follow-up visits. Patients reported headache-related hospital admissions and visits to a healthcare professional (HCP), by type: primary care consultant, outpatient consultation, accident and emergency visit, alternative practitioner, or other. The baseline timeframe reflected the last 3 months before the baseline visit and the follow-up timeframe since the last visit with onabotulinumtoxinA administration. Patients enrolled at German study centres before 08 April 2014 (*n* = 264) completed an additional health economic questionnaire on the use of health services in the 6 months prior to baseline and since the last onabotulinumtoxinA administration visit. Patients were asked about the use of the following services as a result of headaches: 1) visits to a family doctor (general practitioner); 2) visits to a medical specialist (ie, otolaryngologist (ENT), ophthalmologist, neurologist, neurosurgeon, dentist); 3) inpatient treatment in an acute care hospital; 4) technical diagnostics (ie, computed tomography (CT), magnetic resonance imaging (MRI), X-ray, ultrasound); 5) rehabilitation measures; 6) use of remedies (massage, physiotherapy, manual therapy); 7) therapy by an osteopath/non-medical practitioner; 8) acupuncture treatment; 9) nutritional supplements or other over-the-counter supplements used for headache prevention; and 10) medication taken for the acute treatment of headache. The health economic questionnaire also included questions related to incapacity to work, disability, absenteeism from school/work, availability of disabled person’s pass, performance, and life habits (ie, regular endurance sport, regular relaxation exercises).

### Statistical analysis

The analysis population for demographic, HRU, effectiveness, and safety data included all patients who received ≥1 dose of onabotulinumtoxinA (Safety Analysis Set, SAF). Results are presented for the overall study population and stratified by country, when available. The health economic questionnaire data were analysed based on the German Analysis Set, which comprised SAF patients enrolled at German study centres before 08 April 2014. Administration visits were defined as visits at which onabotulinumtoxinA was injected (ie, Admin 1 = baseline, Admin 2 = follow-up visit with second administration, etc.). Administration visit 1 (baseline) data were collected prior to the first onabotulinumtoxinA injection treatment. Data from baseline through administration 8 are reported herein to reflect the expected number of onabotulinumtoxinA treatments administered during a 2-year period according to the SPC.

Changes from baseline in the effectiveness variables were tested using a non-parametric Wilcoxon signed rank test. HRU data was summarized and presented descriptively for the overall study population and by country. Descriptive statistics are presented for continuous variables; frequencies and percentages are provided for categorical data. The McNemar test was used to compare baseline and follow-up data for headache-related hospitalizations and HCP visits for the overall and German populations. For statistical analysis of the German health economic questionnaire data, two-sided 95% confidence intervals (CI) were calculated based on the exact binomial distribution using the Clopper-Pearson method. This methodology signifies a significant difference from baseline when the derived confidence intervals do not overlap. All results are based on available patient data, missing data were not imputed. Statistical analyses were tested at the 2-sided 5% level and conducted with SAS version 9.3 (SAS Institute, Inc., Cary, NC).

## Results

### Study population

A total of 641 patients were enrolled in the REPOSE study and of those patients, 633 received ≥1 onabotulinumtoxinA treatment and were included in the Safety Analysis Set (SAF) and 128 completed 24 months and were included in the Per-Protocol Set (PPS). Of the 633 patients included in the SAF, 144 (22.7%) discontinued treatment. Reasons for treatment discontinuation are listed in Supplemental Table 1. Approximately 60% (*n* = 377) of the patients in the SAF were treated at German study centres and 70% (*n* = 264) of these patients were enrolled before 08 April 2014 and completed the health economic questionnaire. Baseline demographics and clinical characteristics of the overall REPOSE patient population and the German population are presented in Table 1*.* Demographic data by country is provided in Supplemental Table 2. At German study centres, patient baseline characteristics were similar to the overall study population, with a mean (SD) age of 46.3 (11.8) years and the majority of patients female (84.4%, *n* = 318).

**Table 1:** Baseline demographic and migraine characteristics of patients in the REPOSE study (overall and German populations)

	Overall Population^a^ (*n* = 633)	German Population^b^ (*n* = 377)
Mean (SD) age, years	45.4 (11.7)	46.3 (11.8)
Female, n (%)	540 (85.3)	318 (84.4)
Mean (SD) monthly headache days	20.6 (5.4)	18.9 (4.5)

^a^Percentages are based on total number of patients who received ≥1 dosage of onabotulinumtoxinA

^b^Percentages are based on the total number of patients treated at German study centres who received ≥1 dosage of onabotulinumtoxinA

### OnabotulinumtoxinA utilisation

In REPOSE, patients received a mean (SD) of 5.5 (3.0) treatment sessions with onabotulinumtoxinA for CM. OnabotulinumtoxinA utilisation closely followed the SPC with a mean (SD) dose of 155.1 (21.4) U and 31.4 (4.3) injection sites among a mean (SD) number of 6.9 (0.6) muscle areas per session. The median time from baseline to administration visit 8 was 21.7 months. The most frequent deviation from the SPC was a prolongation of the recommended 12-week treatment interval, with 69.5% (*n* = 440/633) of patients receiving ≥1 treatment session 13–16 weeks after the previous session during the observational period.

### Outcome measures

#### Effectiveness

**Fig. 1:**
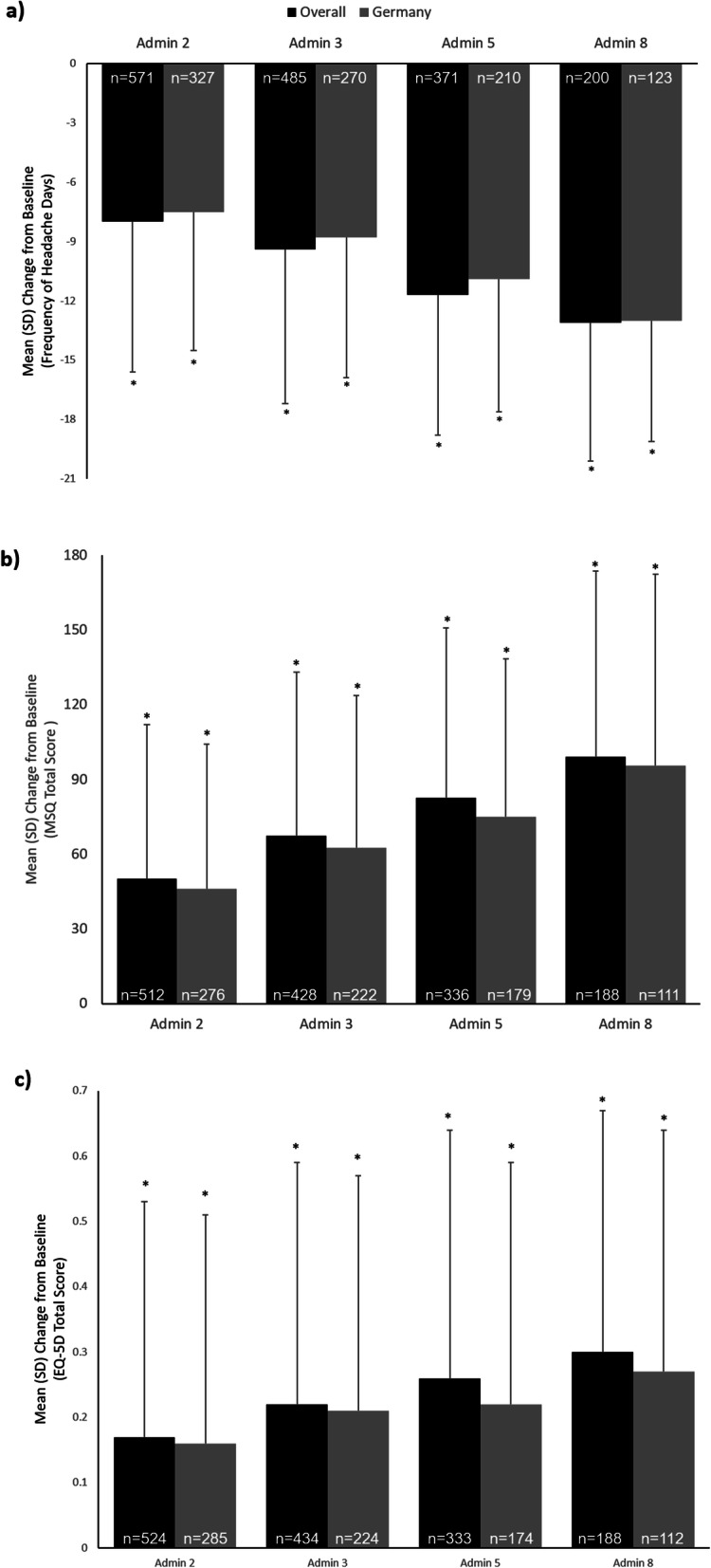
**a** Mean (SD) change from baseline in frequency of headache days. The patient-reported estimate of the number of days in a month with a headache (≥4 h) at each administration visit through visit 8; **b** Mean (SD) change from baseline in total MSQ score; **c** Mean (SD) change from baseline in EQ-5D total score. *P < 0.001 Wilcoxon signed rank test for change versus baseline (level of significance, 5%). Abbreviations: Admin, administration; MSQ = Migraine-Specific Quality-of-Life Questionnaire, EQ-5D = EuroQol 5-Dimension Questionnaire

As recently published in detail by Ahmed et al. [17], long-term, real-world preventive treatment of CM with onabotulinumtoxinA showed effectiveness with a sustained reduction in headache-day frequency and significant improvements in quality of life measures. As presented in Fig. 1a, headache-day frequency was significantly reduced from a baseline mean (SD) of 20.6 (5.4) to 7.4 (6.6) days at administration visit 8 in the overall population and from 18.9 (4.5) to 6.0 (5.8) days in the German population (*p* < 0.001). In both the overall and German populations, total MSQ scores increased significantly at all post-baseline treatment visits, signifying an improvement in patient-reported quality of life across 3 domains [22] (Fig. 1b). Results of the EQ-5D questionnaire also demonstrated a significant improvement in patient-reported quality of life with onabotulinumtoxinA treatment in the overall and German populations, as shown in Fig. 1c. Similar effectiveness outcomes were observed for the other countries, however, statistical analyses were not performed due to small sample sizes (Supplemental Table 3). In addition, the majority of patients and physicians in the overall population and at German study centres rated satisfaction with treatment and treatment tolerability as good or very good throughout the 2-year observation period (Supplemental Figure 1).

#### Healthcare resource utilisation

**Fig. 2:**
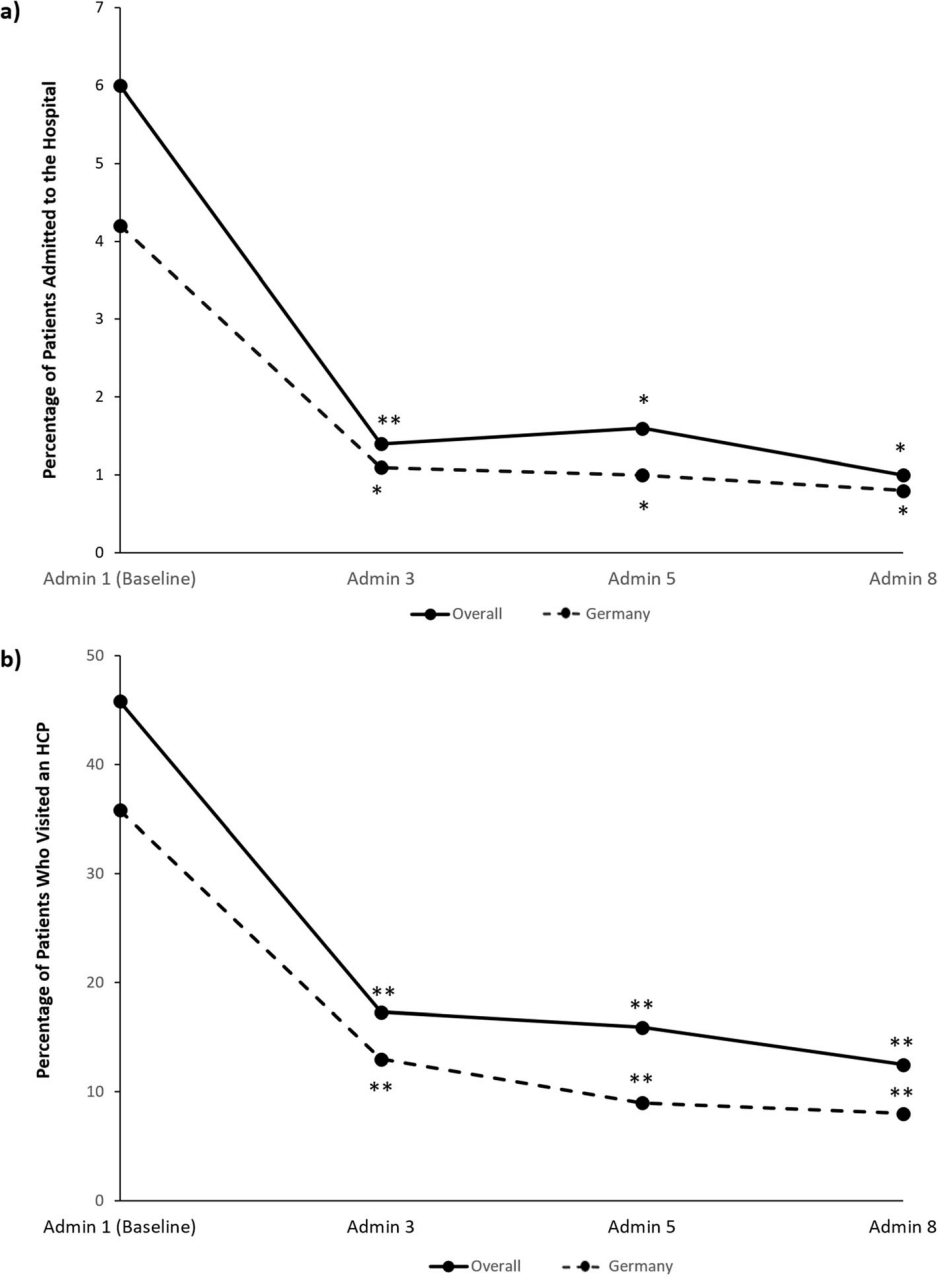
**a** Percentage of patients who reported a headache-related hospitalization in the 3 months prior to baseline or since the last onabotulinumtoxinA administration for follow-up visits; **b** Percentage of patients who visited any HCP in the 3 months prior to baseline or since the last onabotulinumtoxinA administration for follow-up visits. The number of patients in administration (Admin) visits for the overall and German populations are as follows: Admin 1, *n* = 633 overall, *n* = 377 Germany; Admin 3, *n* = 485 overall, *n* = 270 Germany; Admin 5, *n* = 371 overall, *n* = 210 Germany; Admin 8, *n* = 200 overall, *n* = 123 Germany. *P < 0.02, **P < 0.0001 McNemar test for difference versus baseline (level of significance, 5%). Abbreviations: Admin, administration; HCP, healthcare professional

As presented in Fig. 2, HRU decreased significantly from baseline with onabotulinumtoxinA treatment for CM in the overall and German REPOSE study populations. The percentage of patients in the overall population that reported headache-related hospital admissions was significantly reduced from 6.0% (*n* = 38/633) at baseline to 1.7% (*n* = 10/573, *p* = 0.0001) at administration visit 2 and continued to decrease to 1.0% (*n* = 2/200, *p* = 0.005) at administration visit 8. Similar results were seen in the German population, with headache-related hospital admissions reported by 4.2% (*n* = 16/377) of patients at baseline and 0.8% (*n* = 1/123, *p* = 0.02) at administration visit 8 (Fig. 2a). Trends observed within the other countries were consistent with the overall findings though no statistical analyses were performed (Supplemental Table 4). Notably, the percentage of patients reporting headache-related hospitalizations in the 3 months prior to baseline in Italy (15.4%, *n* = 4/26) and Russia (16.1%, *n* = 5/31) decreased to 0% at administration visit 8. In Spain, headache-related hospitalization decreased from 11.4% (*n* = 10/88) at baseline to 3.2% (*n* = 1/31) at administration visit 8.

The proportion of patients who had visited any HCP during the 3 months prior to baseline was 45.8% (*n* = 290/633) in the overall population and 35.8% (*n* = 135/377) in the German population. By administration visit 8, these proportions significantly decreased to 12.5% (*n* = 25/200) in the overall population and 8.1% (*n* = 10/123) in the German population (all *p* < 0.0001). Primary care and outpatient consultations represented the majority of these visits, as shown in Table 2. A statistically significant decrease between baseline and administration visit 8 was observed in both the number of primary care and outpatient consultations in the overall and German populations (*p* < 0.0001). Statistical comparisons regarding the number of accident and emergency visits, visits to alternative practitioners, and visits to other HCPs were not feasible due to the small number of patients who reported such visits.

**Table 2:** Patient-reported visits to healthcare professionals by type of professional

	Admin 1		Admin 3		Admin 5		Admin 8	
	Overall (*n* = 633)	Germany (*n* = 377)	Overall (*n* = 485)	Germany (*n* = 270)	Overall (*n* = 371)	Germany (*n* = 210)	Overall (*n* = 200)	Germany (*n* = 123)
Primary care consultant	194 (30.6)	75 (19.9)	61 (12.6)	21 (7.8)	44 (11.9)	13 (6.2)	19 (9.5)	7 (5.7)
Outpatient consultation	211 (33.3)	95 (25.2)	21 (4.3)	12 (4.4)	22 (5.9)	10 (4.8)	9 (4.5)	5 (4.1)
Accident/emergency visit	40 (6.3)	8 (2.1)	6 (1.2)	2 (0.7)	7 (1.9)	1 (0.5)	3 (1.5)	1 (0.8)
Alternative practitioner	40 (6.3)	21 (5.6)	4 (0.8)	2 (0.7)	3 (0.8)	2 (1.0)	4 (2.0)	3 (2.4)
Other	24 (3.8)	19 (5.0)	9 (1.9)	7 (2.6)	5 (1.3)	3 (1.4)	1 (0.5)	1 (0.8)

Frequencies are presented as n (%)

#### Health economics

The German Analysis Set comprised 264 patients for whom health economics data were collected at German study centres. Results of the health economic questionnaire showed that the health services most frequently used during the 6 months prior to baseline were ‘visit to a medical specialist’ (61.7%, 95% CI [55.6%, 67.6%], *n* = 163/264) and ‘visit to a family doctor’ (41.7%, 95% CI [35.7%, 47.9%], *n* = 110/264). In the 6 months prior to baseline, medication for the acute treatment of headache was taken by 71.2% of patients in the German Analysis Set (*n* = 188/264).

**Fig. 3:**
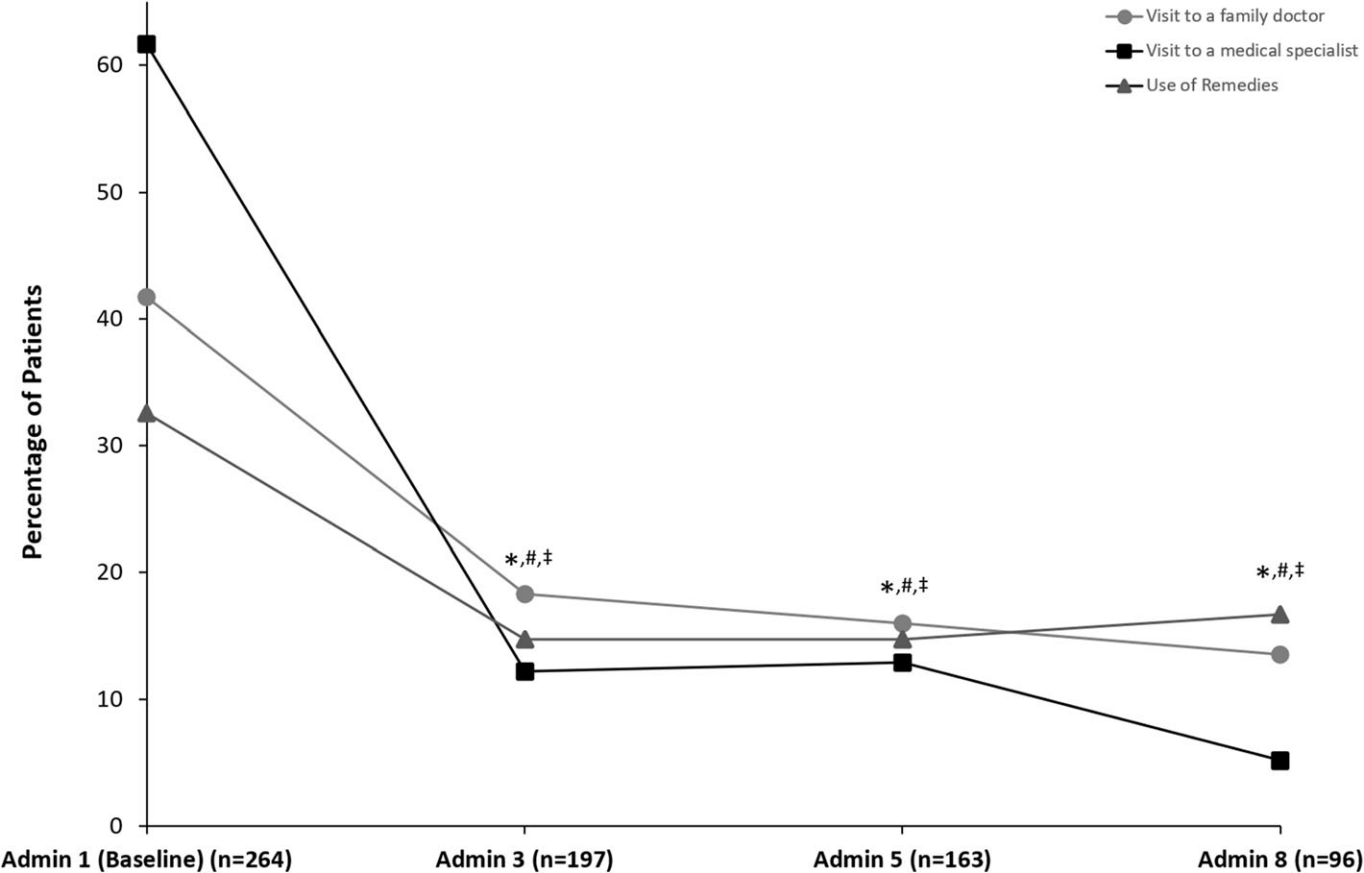
Health economic questionnaire data from German patients in the SAF enrolled before 08 April 2014 showing percentages of patients who visited a family doctor (**●**), visited a medical specialist (■), and used remedies, including massage, manual therapy, and physiotherapy (▲). Percentages are related to the number of patients at the respective visit. The reporting window for the baseline visit was the last 6 months; follow-up since the last visit. Non-overlapping 95% confidence intervals (CIs) at each time point vs. baseline are denoted by * for visits to a family doctor, # for visits to a medical specialist, and ‡ for use of remedies; non-overlapping 95% Cis show statistical significance

At each time point and across all categories, the proportion of patients with health service utilisation since the last visit was less than the proportion of patients with the respective health service utilisation during the 6 months prior to baseline. As presented in Fig. 3, the percentage of patients who reported a visit to a family doctor at administration visit 8 (13.5%, 95% CI [7.4%, 22.0%], *n* = 13/96) was significantly less than the percentage of patients who reported using this service in the 6 months prior to baseline (41.7%, 95% CI [35.7%, 47.9%], *n* = 110/264), as demonstrated by non-overlapping 95% CIs. The percentage of patients reporting a visit to a medical specialist decreased significantly from 61.7% (95% CI [55.6%, 67.6%], *n* = 163/264) at baseline to 5.2% (95% CI [1.7%, 11.7%], *n* = 5/96) at administration 8. The percentage of patients reporting the use of remedies (ie, massage, physiotherapy, and manual therapy) also decreased significantly over the 2-year observation period from 32.6% (95% CI [27.0%, 38.6%], *n* = 86/264) at baseline to 16.7% (95% CI [9.8%, 25.6%], *n* = 16/96) at administration 8. At baseline, the most frequently documented remedy was massage (22.1%, *n* = 57/258), followed by physiotherapy (21.7%, *n* = 56/258) and manual therapy (19.8%, *n* = 51/258). During the 6 months prior to baseline, 8.3% (95% CI [5.3%, 12.3%], *n* = 22/264) of patients received therapy by an osteopath/non-medical practitioner while no patients reported using this service at administration 8 (0%, 95% CI [0%, 3.8%], *n* = 0/96). The percentage of patients who used acupuncture treatment decreased significantly from 14.8% (95% CI [10.7%, 19.6%], *n* = 39/264) at baseline to 2.1% (95% CI [0.3%, 7.3%], *n* = 2/96) at administration 8. Patients also reported a significant decrease in technical diagnostics during the observation period, with 19.7% (95% CI [15.1%, 25.0%], *n* = 52/264) of patients receiving technical diagnostics in the 6 months prior to baseline and only 1.0% (95% CI [0.0%, 5.7%], *n* = 1/96) reporting the use of these services at administration 8. The most frequent examinations reported at baseline were MRI of the head (16.3%, *n* = 42/258) and ultrasound of neck vessels (5.8%, *n* = 15/258). Additionally, significantly fewer patients reported taking dietary supplements for headache prevention since the last administration visit at administration visit 8 (8.3%, 95% CI [3.7%, 15.8%], *n* = 8/96) than in the last 6 months before baseline (17.8%, 95% CI [13.4%, 23.0%], *n* = 47/264).

Fewer patients reported inpatient treatment and acute medication use at administration visit 8 than baseline, though the differences were not statistically significant. At baseline, 6.4% (95% CI [3.8%, 10.1%], *n* = 17/264) of patients reported inpatient treatment in an acute care hospital within the last 6 months. This proportion decreased to 2.6% (95% CI [1.0%, 5.6%], *n* = 6/288) of patients at administration 2, and 0% (95% CI [0%, 3.8%], *n* = 0/96) of patients reported using this service at administration visit 8. Over the 2-year observation period, there was a decrease in the percentage of patients that reported taking medication for the acute treatment of headache, 71.2% (95% CI [65.3%, 76.6%], *n* = 188/264) at baseline and 58.3% (95% CI [47.8%, 68.3%], *n* = 56/96) at administration 8.

**Fig. 4:**
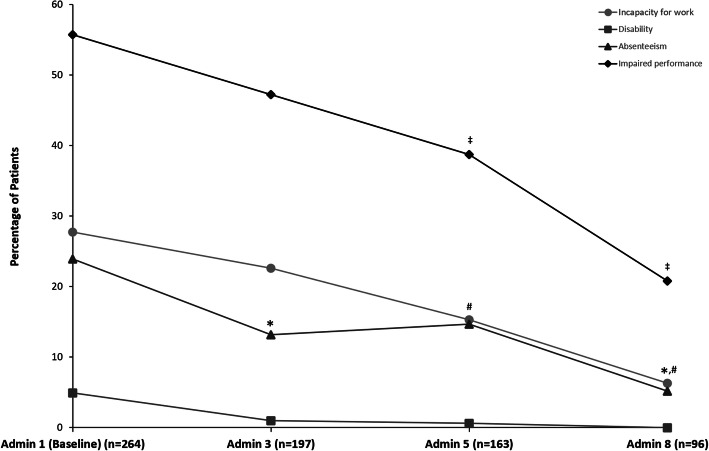
Health economic questionnaire data from German patients in the SAF enrolled before 08 April 2014. Percentage of patients who had been incapacitated for work (**●**) or reported a disability (■) in the last 6 months prior to baseline or since the last visit, and the percentage of patients that had been absent from school or work (▲) or stated that performance at school or work had been impaired when having a headache at school/work (♦) in the last 4 weeks prior to baseline or since the last visit. Percentages are related to the number of patients at the respective visit. Non-overlapping 95% confidence intervals at each time point vs. baseline are denoted by * for absenteeism, # for work incapacity, and ‡ for impaired performance; non-overlapping 95% Cis show statistical significance

Measures of incapacity for work, absenteeism, and impaired performance due to headache improved significantly in the German Analysis Set over the 2-year observation period of onabotulinumtoxinA treatment (Fig. 4). At baseline, 55.7% (95% CI [49.5%, 61.8%], *n* = 147/264) of patients indicated that performance at school or work was impaired when having a headache at school/work in the past 4 weeks whereas 20.8% (95% CI [13.2%, 30.3%], *n* = 20/96) reported this problem at administration 8 since the last administration visit. The percentage of patients that reported absence from school or work during these periods decreased from 23.9% (95% CI [18.9%, 29.5%], *n* = 63/264) at baseline to 5.2% (95% CI [1.7%, 11.7%], *n* = 5/96) at administration 8. In the 6 months prior to baseline, 27.7% (95% CI [22.3%, 33.5%], *n* = 73/264) of patients had been incapacitated for work and a disability was reported by 4.9% (95% CI [2.6%, 8.3%], *n* = 13/264) of patients. The percentage of patients who reported disability also decreased from baseline to administration 8, though the change was not statistically significant. At administration visit 8, the percentage of patients who reported incapacity and disability since the last visit with onabotulinumtoxinA administration decreased to 6.3% (95% CI [2.3%, 13.1%], *n* = 6/96) and 0.0% (95% CI [0.0%, 3.8%], *n* = 0/96), respectively.

### Safety

Adverse drug reactions were reported by 18.3% (*n* = 116/633) of patients in REPOSE, the majority of which were of mild (7.1%, *n* = 45/633) to moderate (7.4%, *n* = 47/633) intensity. ADRs occuring in > 2% of patients included eyelid ptosis (5.4%, *n* = 34/633), neck pain (2.8%, *n* = 18/633), and musculoskeletal stiffness (2.7%, *n* = 17/633). No new safety concerns were identified.

## Discussion

According to the Global Burden of Disease Study, migraine is the second leading cause of years lived with disability [24] and associated with substantial economic burden. Across Europe, CM is associated with higher medical resource use and total costs than episodic migraine [9, 25, 26]. The objective of the REPOSE study was to describe the long-term, real-world use of onabotulinumtoxinA for the symptomatic treatment of adults with CM over a 2-year period, measuring healthcare resource utilisation and patient-reported outcomes observed in clinical practice. Real-world evidence from the REPOSE study demonstrates that long-term treatment with onabotulinumtoxinA is significantly associated with a sustained reduction in monthly headache days, improved quality of life, and decreases in both HRU and work impairment.

In Germany and across all study centres, patient-reported HRU decreased with onabotulinumtoxinA treatment. Significant reductions in headache-related hospital admissions and HCP visits were observed after the first onabotulibumtoxinA administration and both outcomes continued to decline throughout the 2-year observation period. At administration visit 8, headache-related hospital admissions since the last administration visit were reported by only 1% of patients in the overall study population and 0.8% of German patients, compared with 6.0% and 4.2% at baseline, respectively. Headache-related HCP visits were reported by 12.5% of the overall population and 8.1% of German patients at administration visit 8, which was significantly less than the 45.8% of patients overall and 35.8% of German patients who reported HCP visits in the 3 months prior to baseline. Primary care visits and outpatient consultations constituted the majority of HCP visits reported in this study.

Additional HRU data were collected for patients treated at German study centres and showed reductions across all HRU outcomes with onabotulinumtoxinA treatment, including significant decreases in visits to a family doctor, visits to medical specialists, technical diagnostics, and the use of other remedies. Notably, over the 2-year observation period, the percentage of patients who reported visits to a family doctor decreased by 68% and visits to a medical specialist decreased by 92%. Additionally, there was a 49% decrease in the percentage of patients who used remedies such as massage, manual therapy, and physiotherapy for headaches and a 29% decrease in the percentage of patients taking medication for the acute treatment of headaches. In addition to the financial burden that direct medical costs of CM place on individuals and healthcare systems, indirect costs resulting from lost work productivity can also create hardship for individuals with CM and their families [4, 27]. In REPOSE, the percentage of patients who reported incapacity for and absenteeism from school or work decreased significantly by 77% and 78%, respectively, from baseline to administration visit 8. Impaired performance at school or work also decreased significantly by 63% over the 2-year period and no patients reported headache-related disability at administration visit 8. In REPOSE, treatment with onabotulinumtoxinA positively impacted not only headache day frequency but also the direct and indirect costs associated with CM.

Results from the REPOSE study complement a growing body of real-world evidence that demonstrates additional treatment benefits of onabotulinumtoxinA beyond headache day reduction. For instance, in the single-arm, open-label COMPEL study, onabotulinumtoxinA treatment was associated with significant reductions in headache-related HCP visits, emergency room and urgent care visits, and diagnostic tests in adults with CM [28]. Similar reductions in HRU, as well as improvements in workplace productivity, were observed in the multicentre, observational Canadian PREDICT study [29]. A real-world, open-label study by Rothrock et al. [10] of CM patients presenting to a university-based subspecialty headache clinic showed that patients had 55% fewer emergency department visits, 59% fewer urgent care visits, and 57% fewer hospitalizations during the 6-month onabotulinumtoxinA treatment period than in the 6 months before initiating treatment. These reductions in HRU represent a significant cost offset and are consistent with the findings of a study by Naprienko et al. [30] that demonstrated greater cost-savings with onabotulinumtoxinA treatment for CM than topiramate or acupuncture. When compared with oral migraine preventive medications, onabotulinumtoxinA was associated with a significantly lower likelihood of headache-related emergency department visits and hospitalizations in a large, United States healthcare claims database study [31]. Additionally, cost-effectiveness analyses have shown that onabotulinumtoxinA is a cost-effective treatment for chronic migraine in the UK [20, 32], Sweden and Norway [33], and Italy [34].

Collectively, these findings demonstrate the potential for treatment with onabotulinumtoxinA to address the economic burden imposed on individuals and healthcare systems by both the direct and indirect costs of CM. A retrosective observational analysis by Negro et al. [26] of electronic medical records from patients treated at an Italian tertiary headache centre showed that the average annual migraine-related expenditure per patient was €1482, with medications and specialist visits accounting for the majority of expenditures. Furthermore, the annual direct cost of CM was 4.8-fold higher than that of EM (€2037 vs. €427, *p* = 0.001). A recent study by Kikui et al. [35] that aimed to estimate the disease burden of migraine in Japan found that compared with matched controls, migraine patients had higher absenteeism, presenteetism, work productivity impairment, total activity impairment, indirect costs, and more HCP visits in the past 6 months. With considerable variation among healthcare systems worldwide, additional country-specific, real-world data will further inform the economic value of onabotulinumtoxinA treatment for CM in the clinical setting.

The REPOSE study provides long-term, real-world data to support the treatment benefit of onabotulinumtoxinA for CM in clinical practice. No new safety signals were identified with longer-term use and administration using real-world prescribing patterns. The REPOSE study population is representative of the typical migraine population and included study centres across 7 countries. The results are generalizable to routine clinical practice in these countries, though sample sizes were small in some participating countries. All real-world studies are subject to some limitations due to their observational nature, including recall bias, loss of follow-up, and the lack of formal protocol requirements and exclusion criteria. When interpreting these data, it is important to note that the results represent real-world treatment conditions where patients may have been taking (including stopping/starting treatment; dose decrease/increase) concomitant preventive medications. Additionally, onabotulinumotxinA treatment disconitnuation in REPOSE due to lack of efficacy may have resulted in an enriched patient population that could potentially confound the results. The number of patients who reported HRU was small especially at later visits, which may reflect a population of patients who responded to treatment, and therefore, the results should be interpreted accordingly.

## Conclusions

CM treatment with onabotulinumtoxinA was associated with a significant reduction in monthly headache days and decreased HRU in German patients, including headache-related hospitalizations, visits to a family doctor, visits to a medical specialist, technical diagnostics, and the use of other remedies. These data support the long-term benefits associated with the use of onabotulinumtoxinA for the treatment of CM in German clinical practice.

## Supplementary Information

**Additional file 1: Supplemental Table 1.** End of study reasons and reasons for discontinuation.

**Additional file 2: Supplemental Table 2.** Baseline demographics, migraine history, and clinical characteristics of patients in the REPOSE study (overall and by country)^a^.

**Additional file 3: Supplemental Table 3.** Change from baseline in effectiveness outcomes: MSQ v2.1 and EQ-5D, by country.

**Additional file 4: Supplemental Table 4.** Percentage of patients who reported headache-related hospitalizations and HCP visits in the 3 months prior to baseline or since the last onabotulinumtoxinA administration for follow-up visits, by country.

**Additional file 5: Supplemental Figure 1. A)** Physician (left) and patient (right) satisfaction with onabotulinumtoxinA treatment in the overall REPOSE study population **B)** Physician (left) and patient (right) satisfaction with onabotulinumtoxinA treatment in the German study population **C)** Physician (left) and patient (right) evaluation of onabotulinumtoxinA treatment tolerability in the overall REPOSE study population **D)** Physician (left) and patient (right) evaluation of onabotulinumtoxinA treatment tolerability in the REPOSE study population.

## Data Availability

AbbVie is committed to responsible data sharing regarding the clinical trials we sponsor. This includes access to anonymized, individual and trial-level data (analysis data sets), as well as other information (e.g., protocols and Clinical Study Reports), as long as the trials are not part of an ongoing or planned regulatory submission. This includes requests for clinical trial data for unlicensed products and indications. This clinical trial data can be requested by any qualified researchers who engage in rigorous, independent scientific research, and will be provided following review and approval of a research proposal and Statistical Analysis Plan (SAP) and execution of a Data Sharing Agreement (DSA). Data requests can be submitted at any time and the data will be accessible for 12 months, with possible extensions considered. For more information on the process, or to submit a request, visit the following link: https://www.abbvie.com/our-science/clinical-trials/clinical-trials-data-and-information-sharing/data-and-information-sharing-with-qualified-researchers.html.

## Abbreviations

CI: Confidence interval
CM: Chronic migraine
EQ-5D: EuroQol 5-Dimension Questionnaire
HCP: Healthcare professional
HRU: Healthcare resource utilization
MSQ: Migraine Specific Quality of Life Questionnaire
PASS: Post-Authorization Safety Study
PREEMPT: Phase III Research Evaluating Migraine Prophylaxis Therapy
SAF: Safety analysis set
SD: Standard deviation
SPC: Summary of product characteristics
