# Supplementary material for: Real-life use of onabotulinumtoxinA reduces healthcare resource utilization in individuals with chronic migraine: the REPOSE study
Source: J Headache Pain. 2021 Jun 2;22(1):50. doi: 10.1186/s10194-021-01260-4 (PMC8173963; doi:10.1186/s10194-021-01260-4)
Supplement: Supplementary file 1 — Additional file 1: Supplemental Table 1. End of study reasons and reasons for discontinuation. [file 10194_2021_1260_MOESM1_ESM.docx]

**Supplemental** **Table 1.** End of study reasons and reasons for discontinuation

| **Reason** | **SAF (N=633)** |
| --- | --- |
| **End of study reasons [n (%)]^a^:** |  |
| No scheduled visits for a future treatment within the study period | 402 (63.5%) |
| Patient will discontinue onabotulinumtoxinA treatment | 144 (22.7%) |
| Patient will continue treatment, but does not wish to continue to participate in the study | 41 (6.5%) |
| End of study reason was not provided | 46 (7.3%) |
| **Reasons for discontinuation of onabotulinumtoxinA treatment [n (%)]^a,b^:** |  |
| Lack of efficacy^c^ | 90 (14.2%) |
| Patient thinks it is inconvenient to come | 17 (2.7%) |
| Side effect(s) or other health problems related to treatment^c^ | 15 (2.4%) |
| Patient thinks injections are too painful | 8 (1.3%) |
| Patient is concerned about risk | 6 (0.9%) |
| Patient thinks the injections take too much time | 2 (0.3%) |
| Patient was trying to get pregnant or was nursing during the study | 2 (0.3%) |
| Patient thinks the treatment is too expensive | 0 |
| Reason was not provided (missing) | 1 (0.2%) |
| Other | 36 (5.7%) |
| ^a^ Percentages are based on the total number of patients in the SAF.  ^b^ More than one reason for discontinuation may have been reported per patient.  ^c^ According to physician’s and/or patient’s assessment | |
