# Supplementary material for: Real-life use of onabotulinumtoxinA reduces healthcare resource utilization in individuals with chronic migraine: the REPOSE study
Source: J Headache Pain. 2021 Jun 2;22(1):50. doi: 10.1186/s10194-021-01260-4 (PMC8173963; doi:10.1186/s10194-021-01260-4)
Supplement: Supplementary file 2 — Additional file 2: Supplemental Table 2. Baseline demographics, migraine history, and clinical characteristics of patients in the REPOSE study (overall and by country)a. [file 10194_2021_1260_MOESM2_ESM.docx]

**Supplemental Table 2.** Baseline demographics, migraine history, and clinical characteristics of patients in the REPOSE study (overall and by country)^a^

|  | Overall  (n=633) | Germany  (n=377) | UK  (n=94) | Italy (n=26) | Spain (n=88) | Norway/  Sweden (n=17) | Russia (n=31) |
| --- | --- | --- | --- | --- | --- | --- | --- |
| Mean (SD) age, years | 45.4 (11.7) | 46.3 (11.8) | 45.2 (12.4) | 46.8 (10.9) | 43.6 (10.5) | 43.7 (12.2) | 39.8 (10.6) |
| Female, n (%) | 540 (85.3) | 318 (84.4) | 81 (86.2) | 23 (88.5) | 77 (87.5) | 15 (88.2) | 26 (83.9) |
| Mean (SD) monthly headache days | 20.6 (5.4) | 18.9 (4.5) | 25.0 (5.8) | 22.8 (5.7) | 21.5 (5.0) | 20.6 (5.2) | 24.5 (4.9) |

UK, United Kingdom

^a^Percentages are based on total number of patients who received ≥1 dosage of onabotulinumtoxinA
