# Supplementary material for: Real-life use of onabotulinumtoxinA reduces healthcare resource utilization in individuals with chronic migraine: the REPOSE study
Source: J Headache Pain. 2021 Jun 2;22(1):50. doi: 10.1186/s10194-021-01260-4 (PMC8173963; doi:10.1186/s10194-021-01260-4)
Supplement: Supplementary file 3 — Additional file 3: Supplemental Table 3. Change from baseline in effectiveness outcomes: MSQ v2.1 and EQ-5D, by country. [file 10194_2021_1260_MOESM3_ESM.docx]

**Supplemental Table 3.** Change from baseline in effectiveness outcomes: MSQ v2.1 and EQ-5D, by country

| **Mean (SD) change from baseline** | **Overall Population**  **N=633** | **Germany**  **N=377** | **UK**  **N=94** | **Italy**  **N=26** | **Spain**  **N=88** | **Norway/Sweden**  **N=17** | **Russia**  **N=31** |
| --- | --- | --- | --- | --- | --- | --- | --- |
| **MSQ v2.1** |  |  |  |  |  |  |  |
| Admin 3 | 67.3 (65.7) | 62.5 (61.3) | 68.0 (74.8) | 57.7 (75.0) | 74.1 (59.8) | 51.5 (61.7) | 104.3 (77.7) |
| Admin 5 | 82.7 68.312 | 75.1 (63.4) | 98.1 (84.3) | 75.0 (63.8) | 85.6 (65.0) | 106.1 (45.2) | 101.8 (91.1) |
| Admin 8 | 99.1 (74.6) | 95.5 (77.1) | 110.9 (73.4) | 84.9 (76.1) | 103.6 (68.7) | 102.1 (56.4) | 116.2 (99.9) |
| **EQ-5D** |  |  |  |  |  |  |  |
| Admin 3 | 0.22 (0.37) | 0.21 (0.36) | 0.14 (0.33) | 0.12 (0.34) | 0.32 (0.40) | 0.13 (0.34) | 0.42 (0.36) |
| Admin 5 | 0.26 (0.38) | 0.22 (0.37) | 0.18 (0.32) | 0.17 (0.33) | 0.42 (0.38) | 0.10 (0.39) | 0.43 (0.35) |
| Admin 8 | 0.30 (0.37) | 0.27 (0.37) | 0.34 (0.36) | 0.24 (0.27) | 0.43 (0.37) | 0.22 (0.31) | 0.26 (0.56) |

Admin, administration visit; EQ-5D, EuroQol 5 Dimension Questionnaire; MSQ, Migraine-Specific Quality of Life Questionnaire
