# Supplementary material for: Real-life use of onabotulinumtoxinA reduces healthcare resource utilization in individuals with chronic migraine: the REPOSE study
Source: J Headache Pain. 2021 Jun 2;22(1):50. doi: 10.1186/s10194-021-01260-4 (PMC8173963; doi:10.1186/s10194-021-01260-4)
Supplement: Supplementary file 4 — Additional file 4: Supplemental Table 4. Percentage of patients who reported headache-related hospitalizations and HCP visits in the 3 months prior to baseline or since the last onabotulinumtoxinA administration for follow-up visits, by country. [file 10194_2021_1260_MOESM4_ESM.docx]

**Supplemental Table 4.** Percentage of patients who reported headache-related hospitalizations and HCP visits in the 3 months prior to baseline or since the last onabotulinumtoxinA administration for follow-up visits, by country

| **Percentage of patients, n (%)** | **Overall Population**  **N=633** | **Germany**  **N=377** | **UK**  **N=94** | **Italy**  **N=26** | **Spain**  **N=88** | **Norway/Sweden**  **N=17** | **Russia**  **N=31** |
| --- | --- | --- | --- | --- | --- | --- | --- |
| **Hospitalization** |  |  |  |  |  |  |  |
| Admin 1 (baseline) | 38 (6.0) | 16 (4.2) | 2 (2.1) | 4 (15.4) | 10 (11.4) | 1 (5.9) | 5 (16.1) |
| Admin 3 | 7 (1.4) | 3 (1.1) | 2 (2.7) | 1 (4.3) | 1 (1.3) | 0 | 0 |
| Admin 5 | 6 (1.6) | 2 (1.0) | 1 (2.0) | 3 (18.8) | 0 | 0 | 0 |
| Admin 8 | 2 (1.0) | 1 (0.8) | 0 | 0 | 1 (3.2) | 0 | 0 |
| **HCP Visit** |  |  |  |  |  |  |  |
| Admin 1 (baseline) | 290 (45.8) | 135 (35.8) | 64 (68.1) | 4 (15.4) | 58 (65.9) | 10 (58.8) | 19 (61.3) |
| Admin 3 | 84 (17.3) | 35 (13.0) | 20 (27.4) | 0 | 22 (28.2) | 4 (26.7) | 3 (11.5) |
| Admin 5 | 59 (15.9) | 19 (9.0) | 17 (34.0) | 0 | 17 (25.0) | 2 (18.2) | 4 (25.0) |
| Admin 8 | 25 (12.5) | 10 (8.1) | 7 (31.8) | 0 | 7 (22.6) | 1 (11.1) | 0 |
