# Supplementary material for: Real-life use of onabotulinumtoxinA reduces healthcare resource utilization in individuals with chronic migraine: the REPOSE study
Source: J Headache Pain. 2021 Jun 2;22(1):50. doi: 10.1186/s10194-021-01260-4 (PMC8173963; doi:10.1186/s10194-021-01260-4)
Supplement: Supplementary file 5 — Additional file 5: Supplemental Figure 1. A) Physician (left) and patient (right) satisfaction with onabotulinumtoxinA treatment in the overall REPOSE study population B) Physician (left) and patient (right) satisfaction with onabotulinumtoxinA treatment in the German study population C) Physician (left) and patient (right) evaluation of onabotulinumtoxinA treatment tolerability in the overall REPOSE study population D) Physician (left) and patient (right) evaluation of onabotulinumtoxinA treatment tolerability in the REPOSE study population. [file 10194_2021_1260_MOESM5_ESM.docx]

**Supplemental Figure 1.**

**
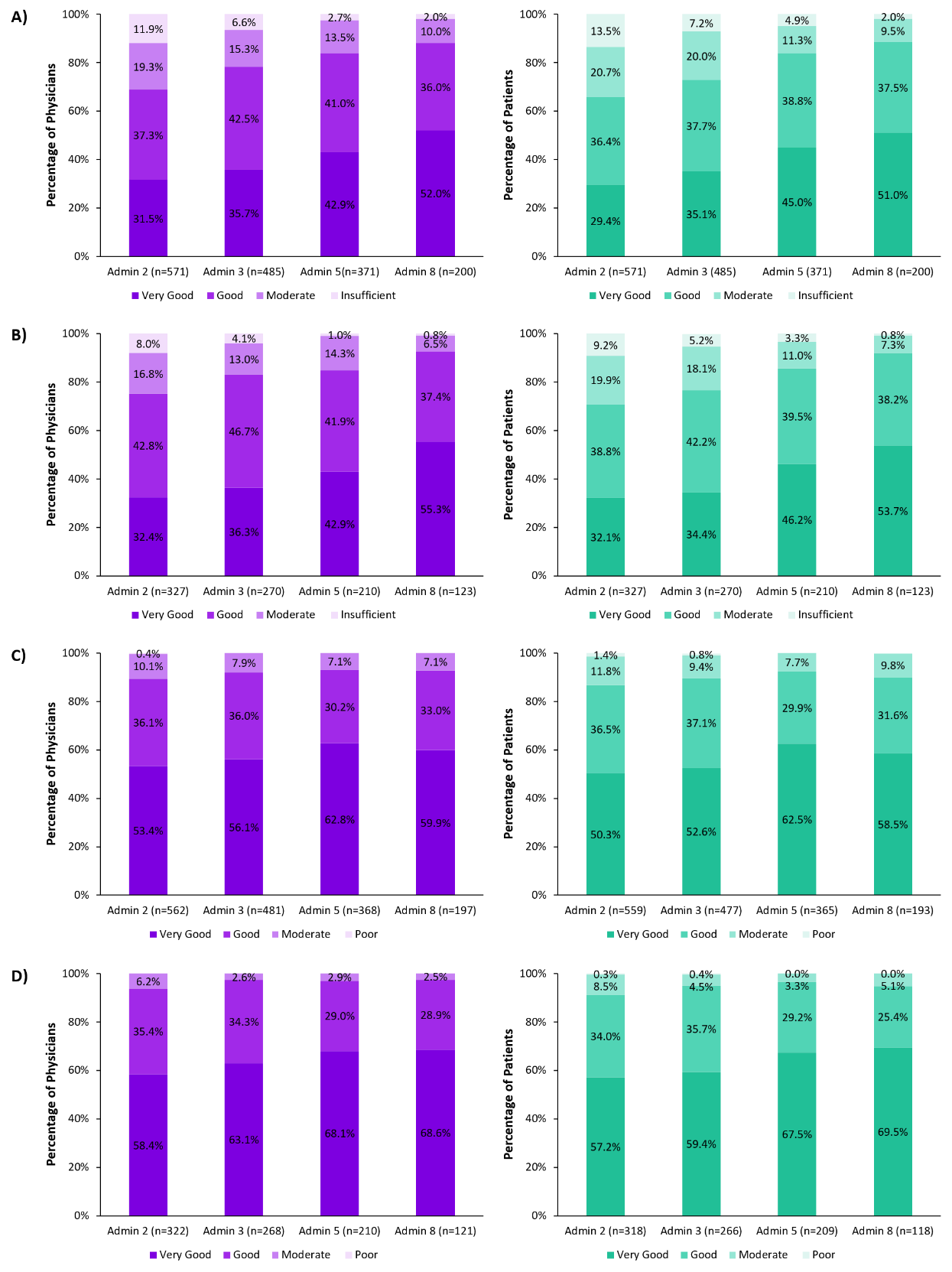
**

**A)** Physician (left) and patient (right) satisfaction with onabotulinumtoxinA treatment in the overall REPOSE study population **B)** Physician (left) and patient (right) satisfaction with onabotulinumtoxinA treatment in the German study population **C)** Physician (left) and patient (right) evaluation of onabotulinumtoxinA treatment tolerability in the overall REPOSE study population **D)** Physician (left) and patient (right) evaluation of onabotulinumtoxinA treatment tolerability in the REPOSE study population
